# Supplementary figures and images for: TRPS1 modulates chromatin accessibility to regulate estrogen receptor alpha (ER) binding and ER target gene expression in luminal breast cancer cells
Source: PLoS Genet. 2024 Feb 20;20(2):e1011159. doi: 10.1371/journal.pgen.1011159 (PMC10906895; doi:10.1371/journal.pgen.1011159)

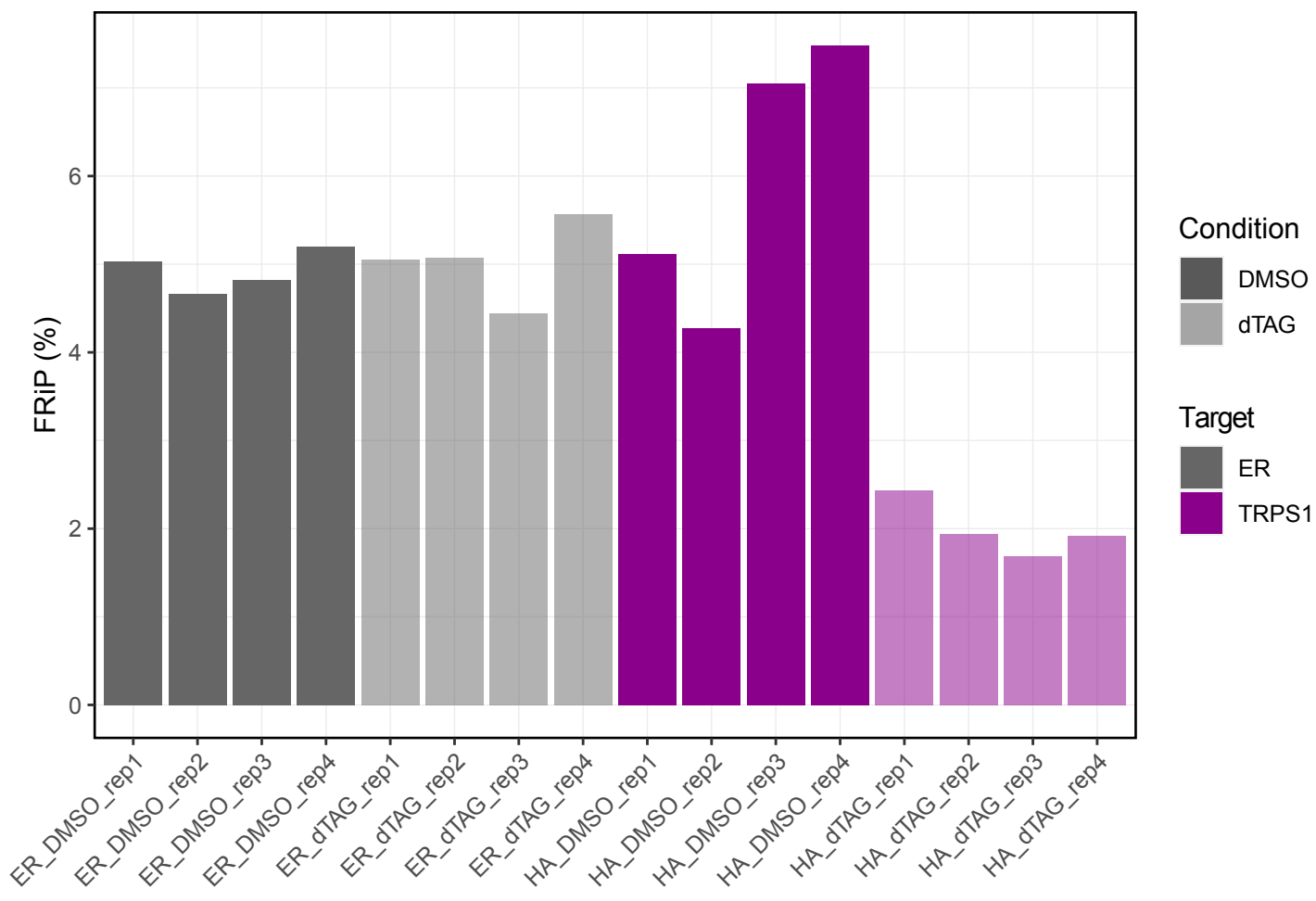

Supplement: S1 Fig — FRiP scores for each library, calculated using the ChIPQC R package [34]. (PDF) [file pgen.1011159.s001.pdf]

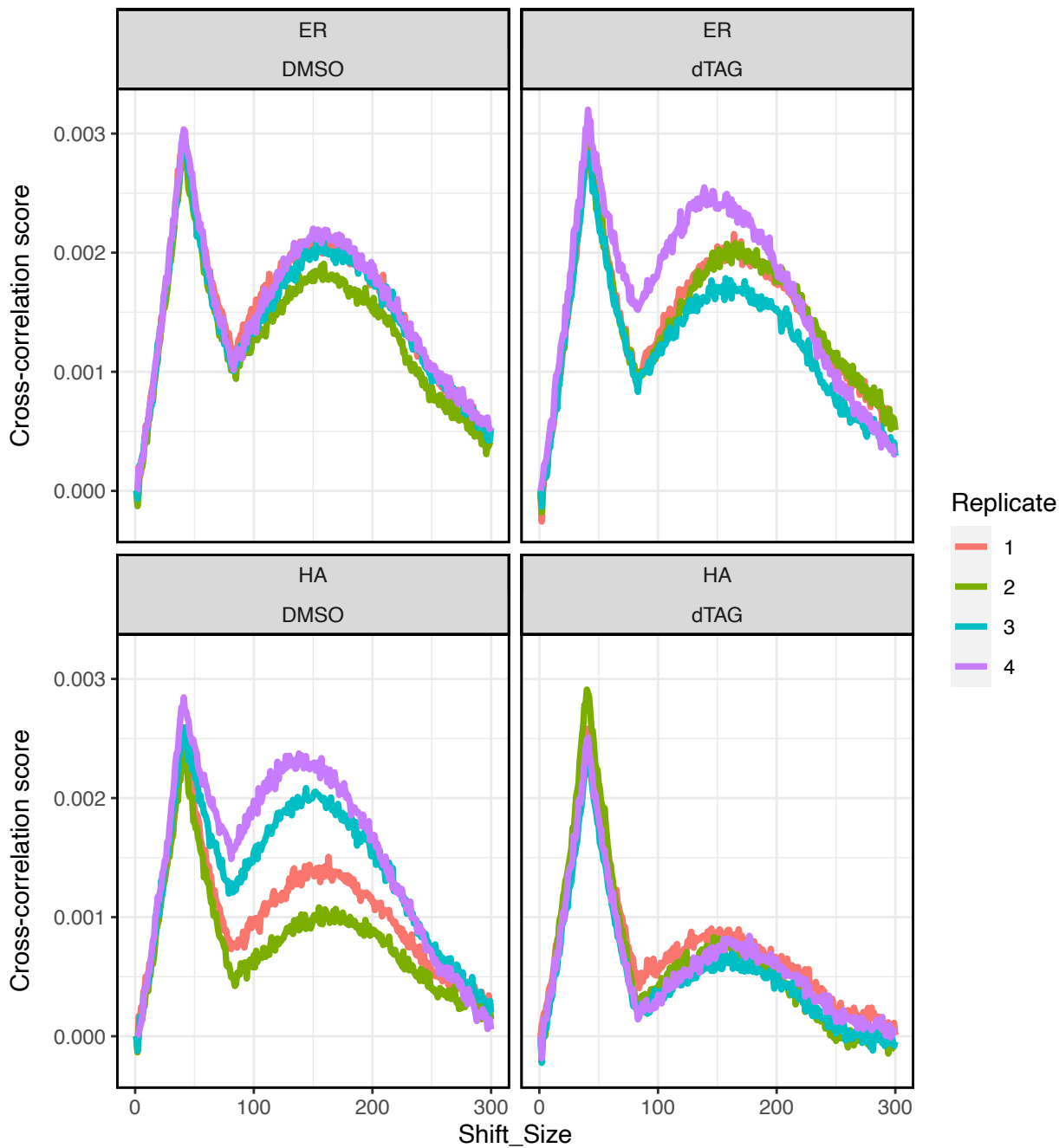

Supplement: S2 Fig — CC values for each library, calculated using the ChIPQC R package [34]. (PDF) [file pgen.1011159.s002.pdf]

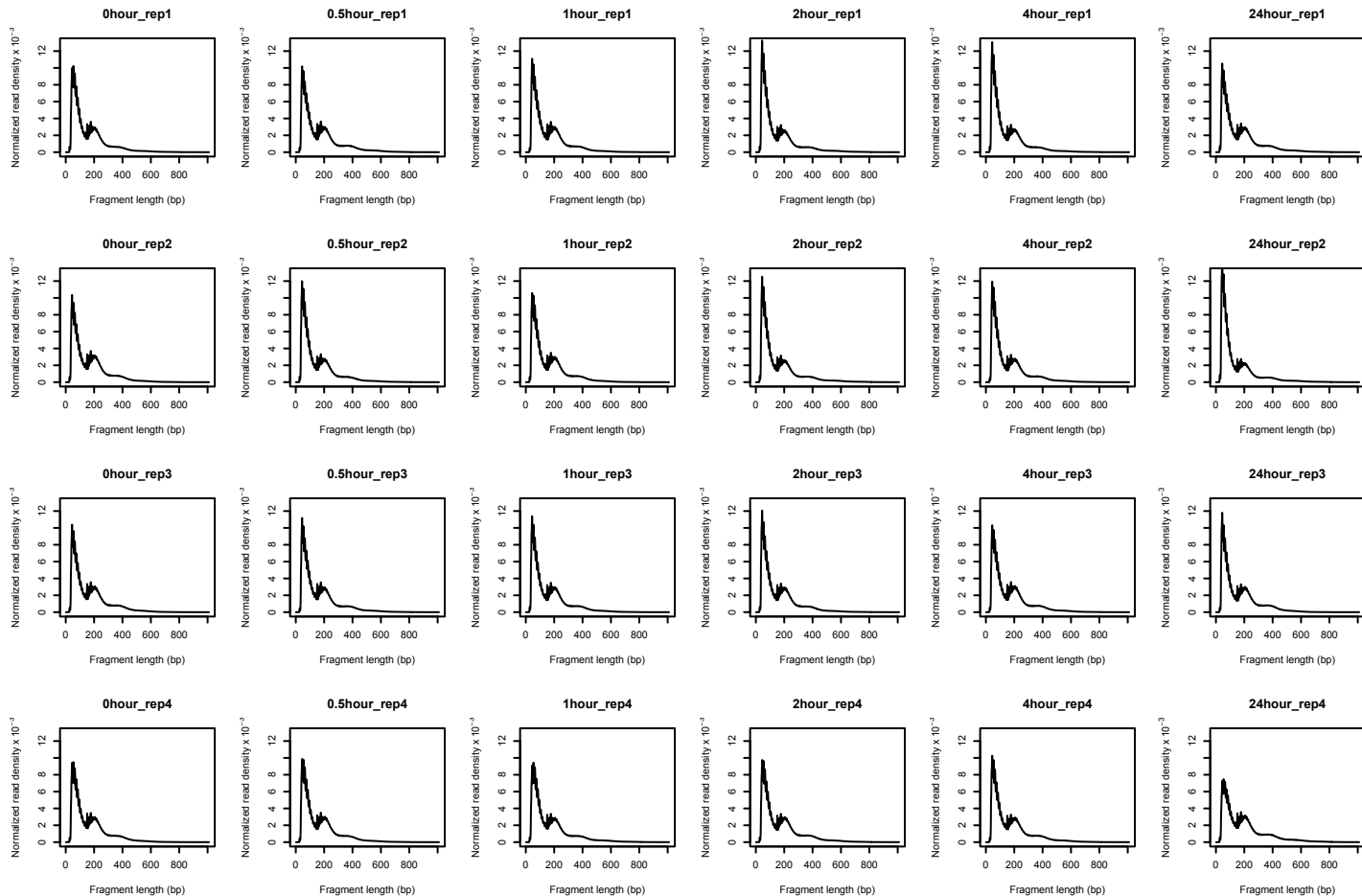

Supplement: S3 Fig — A plot for each library was generated using the ATACseqQC R package [36]. (PDF) [file pgen.1011159.s003.pdf]

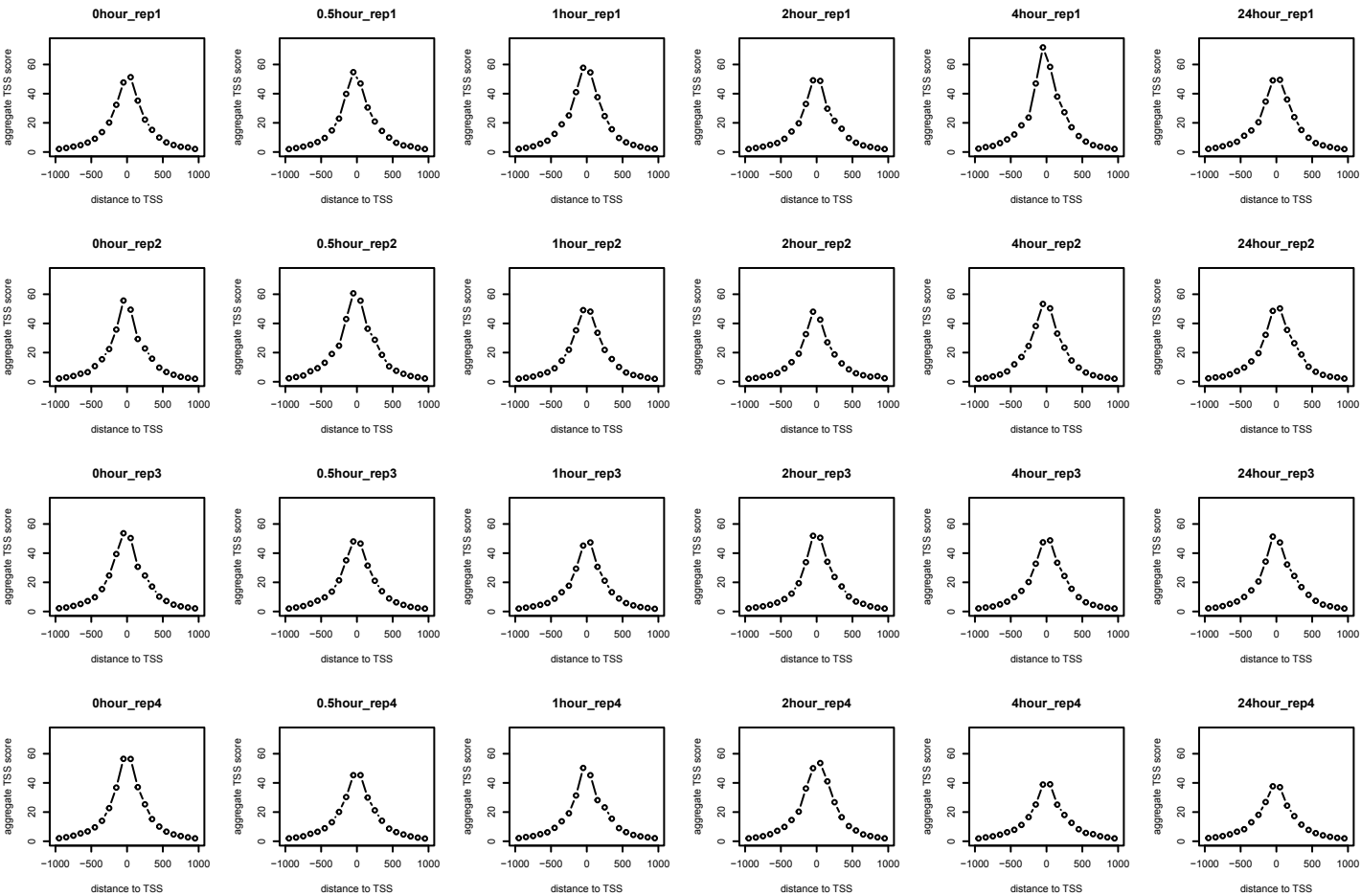

Supplement: S4 Fig — A plot for each library was generated using the ATACseqQC R package [36]. (PDF) [file pgen.1011159.s004.pdf]

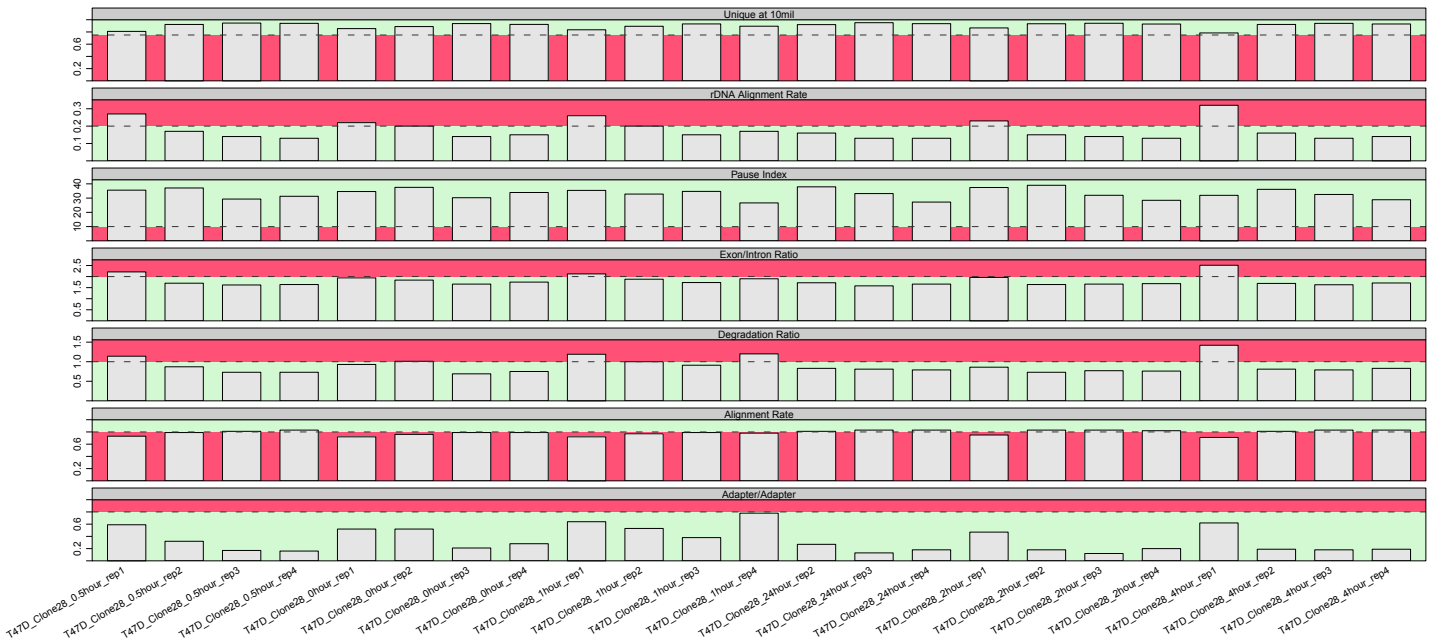

Supplement: S5 Fig — Quality control metrics are defined as in [39]. Each metric is a row, and each sample is a column. The green region for each metric is the goal for a high quality library. (PDF) [file pgen.1011159.s005.pdf]

# Change in bidirectional transcription at TRPS1 peaks 30 minutes after TRPS1 depletion

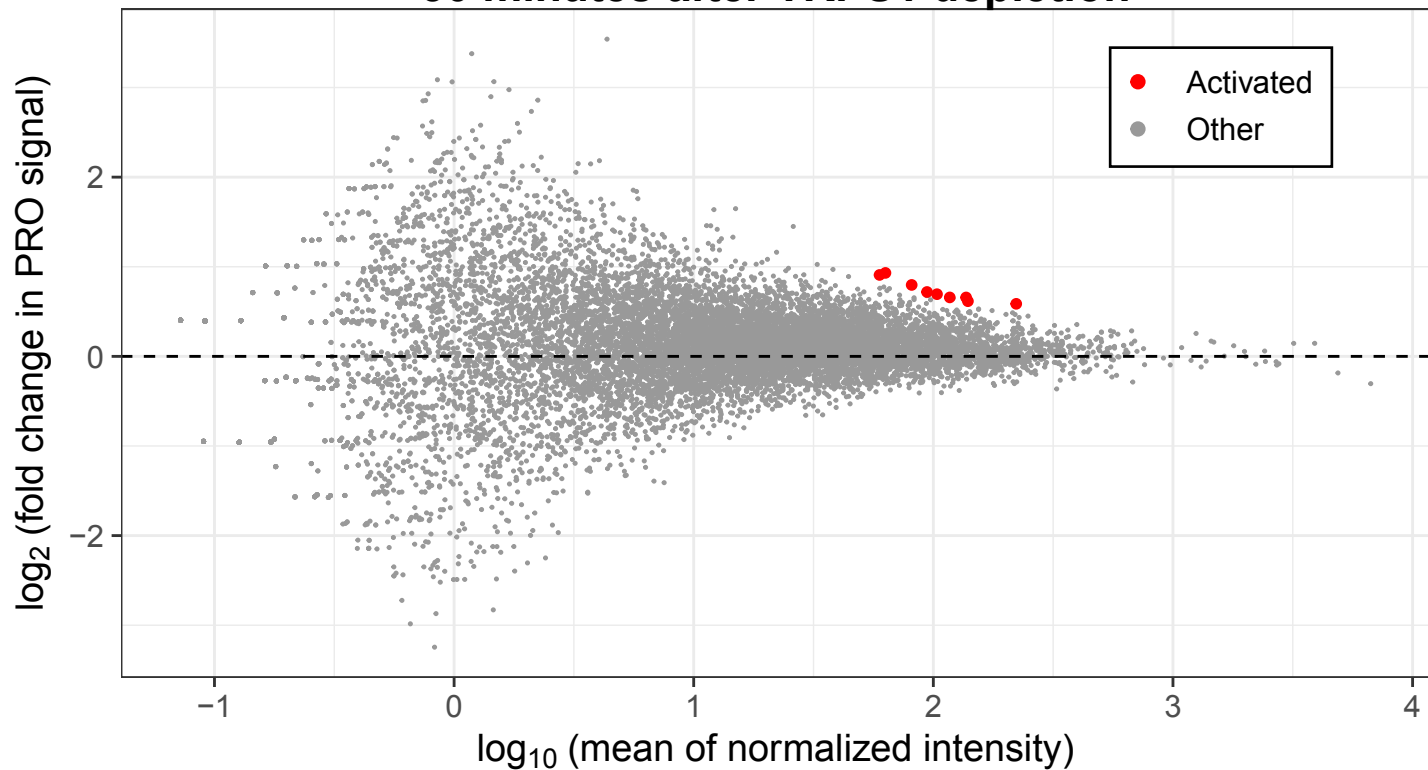

Supplement: S6 Fig — MA plot of TRPS1 ChIP-seq peaks from Fig 3G, with fold change values representing bidirectional transcription in the 30 minute dTAG-13 and dTAGV -1 at 50nM each (dTAG) treatment condition relative to the DMSO condition. Testing for a TRPS1 cistrome-wide increase in bidirectional transcription, the ANOVA F-test p-value was < 2.2*10-16. (PDF) [file pgen.1011159.s006.pdf]

# Change in chromatin accessibility upon TRPS1 depletion distal to dREG elements

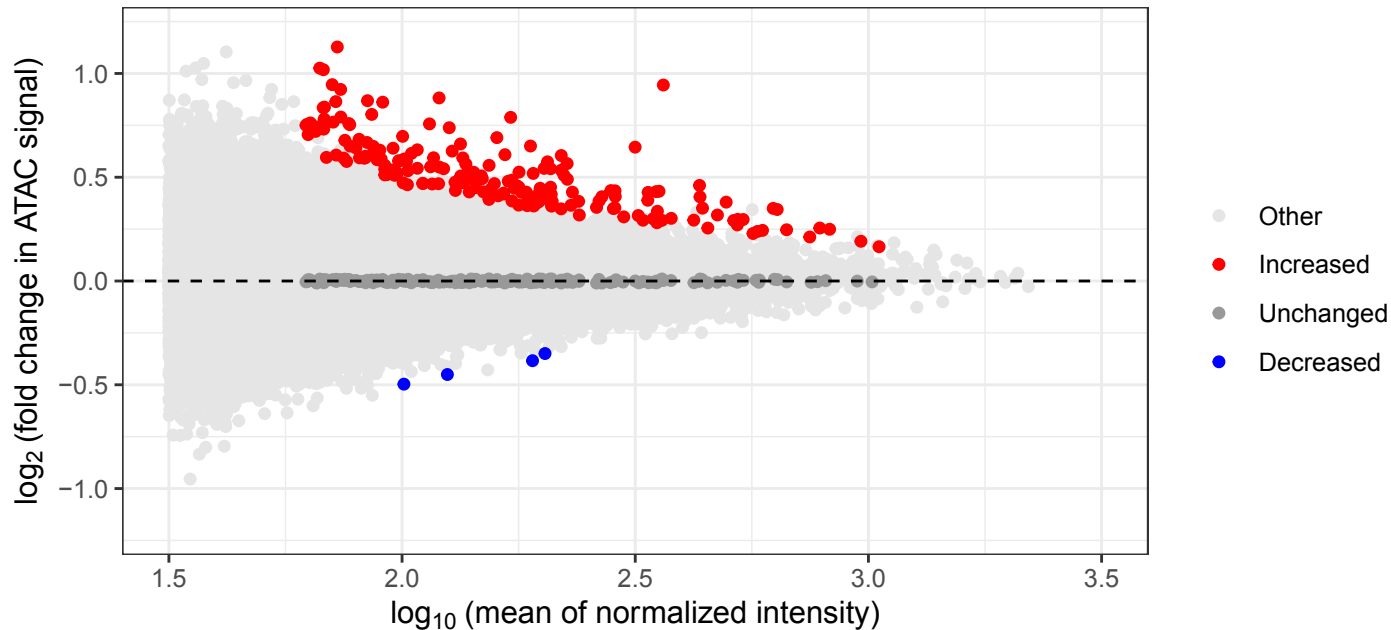

Supplement: S7 Fig — MA plot of ATAC-seq peaks, with fold change values representing accessibility in the 30 minute dTAG-13 and dTAGV -1 at 50nM each (dTAG) treatment condition relative to the DMSO condition, as in Fig 3. (PDF) [file pgen.1011159.s007.pdf]

# Change in gene expression 90 minutes after estrogen treatment

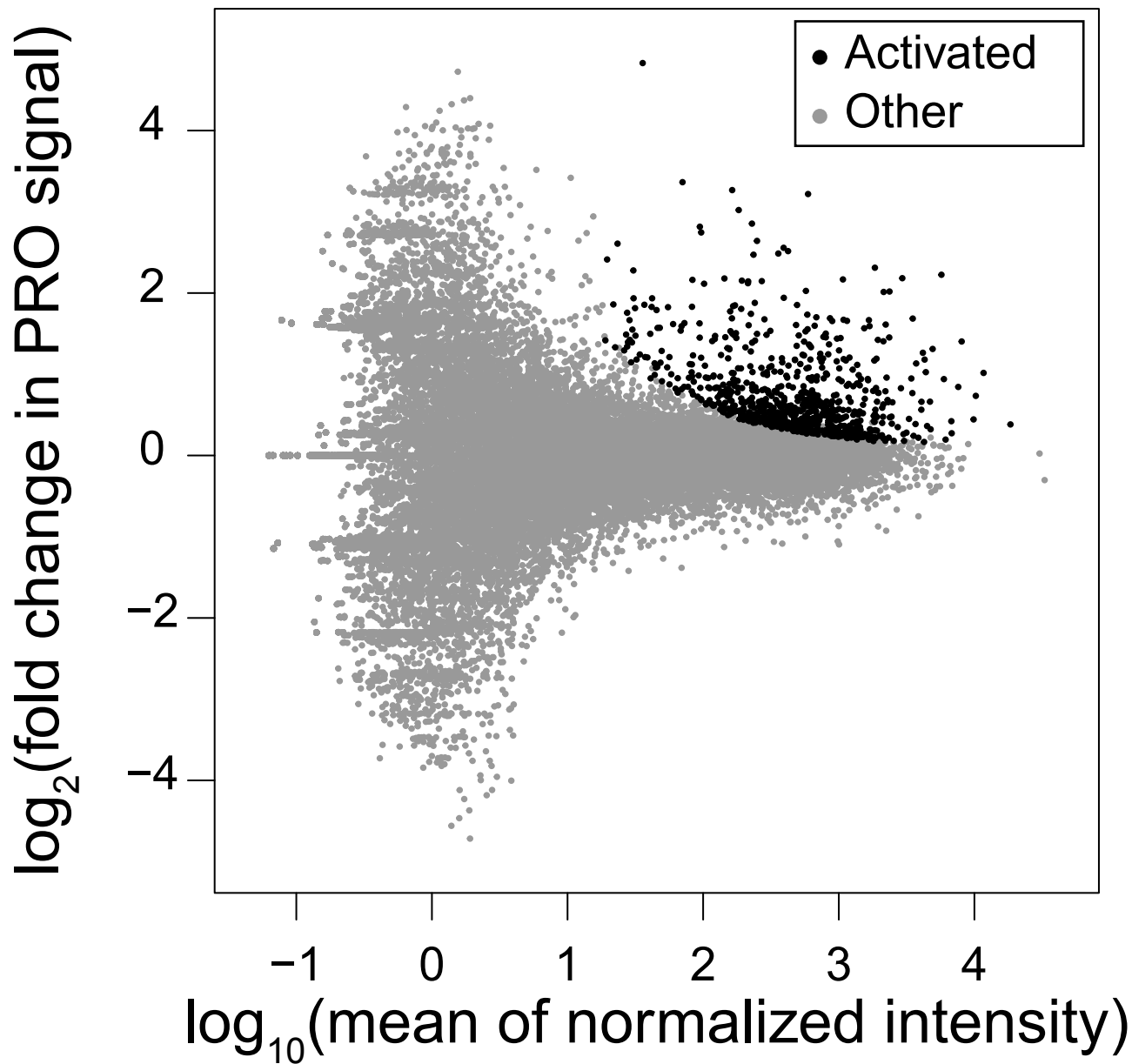

Supplement: S8 Fig — MA plot of PRO signal, with fold change values representing transcription in the 90 minute estrogen treatment condition relative to the DMSO condition. Each point represents a gene, and black points represent the estrogen-activated genes that we use in Fig 5. (PDF) [file pgen.1011159.s008.pdf]

**A**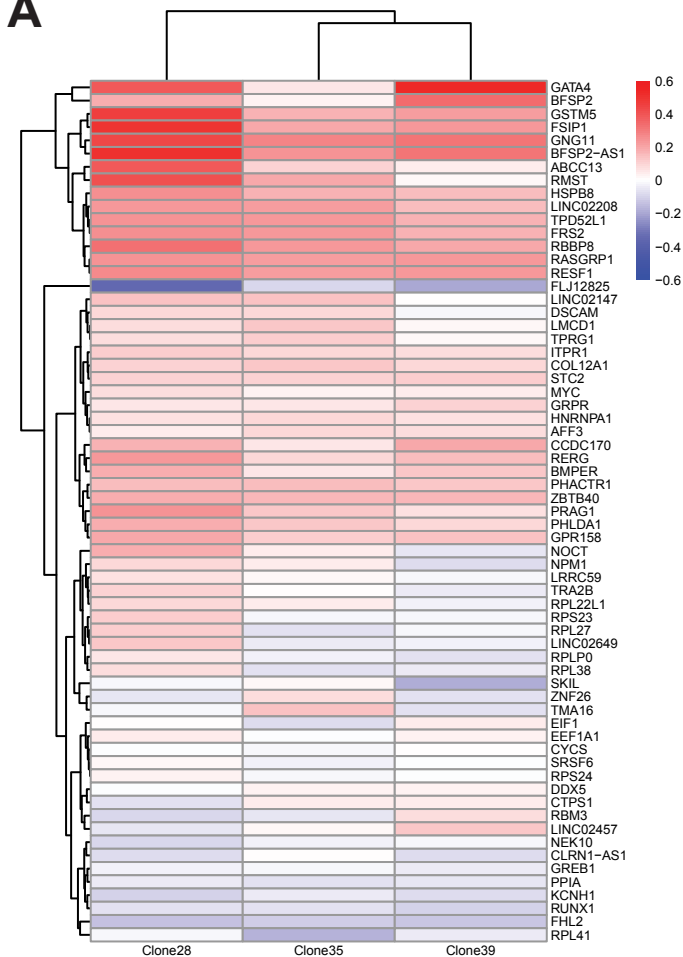**B**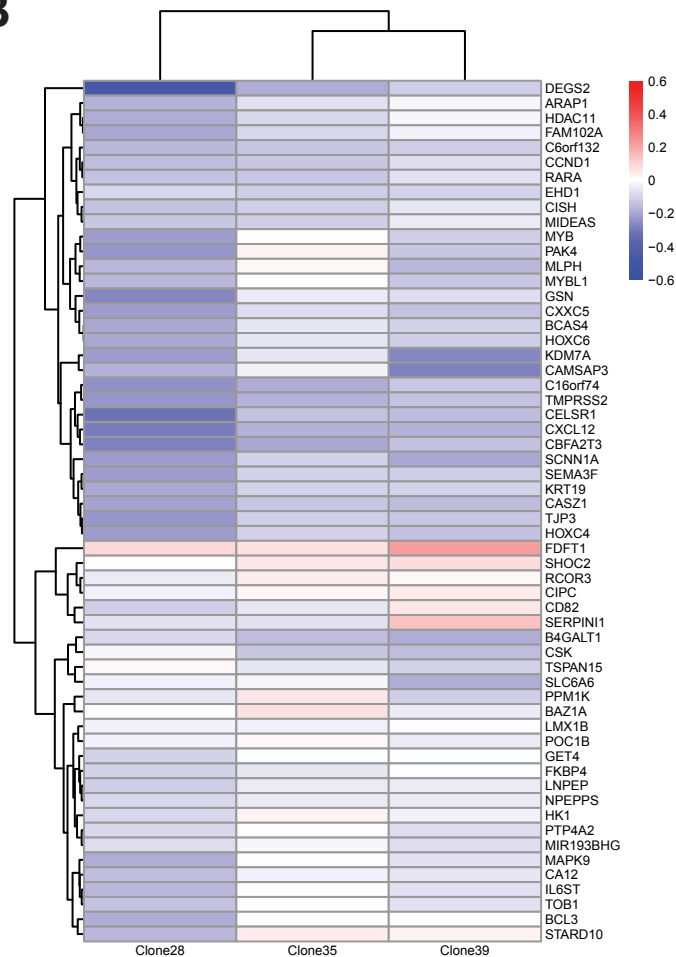

Supplement: S9 Fig — Fold changes in normalized PRO signal across three independent clones for ER target genes that are (A) activated or (B) repressed upon TRPS1 depletion, as defined in Fig 5A. (PDF) [file pgen.1011159.s009.pdf]

# Change in cell number upon TRPS1 depletion

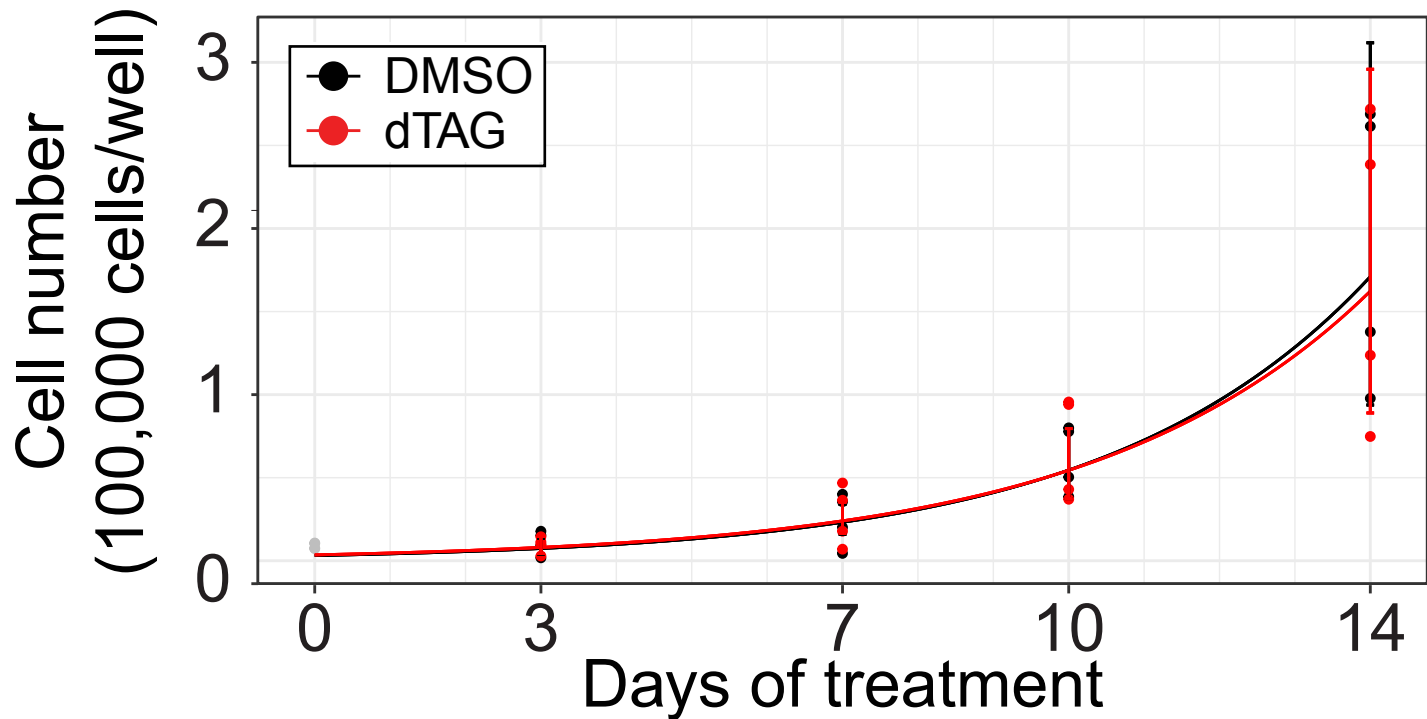

Supplement: S10 Fig — Cell number over time of parental T47D cells treated with dTAG or DMSO, as in Fig 6C. (PDF) [file pgen.1011159.s010.pdf]

**A****ER-positive**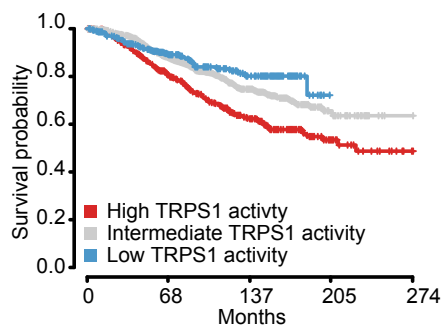**ER-negative**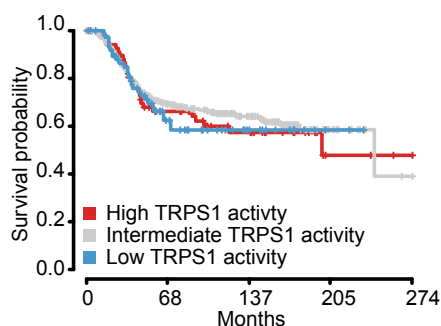**B****Luminal A**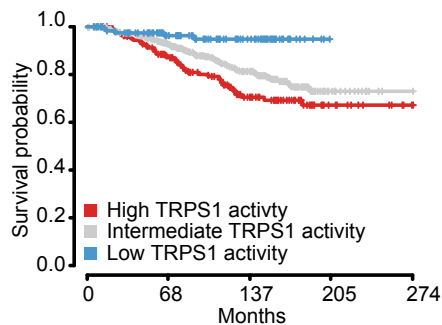**Luminal B**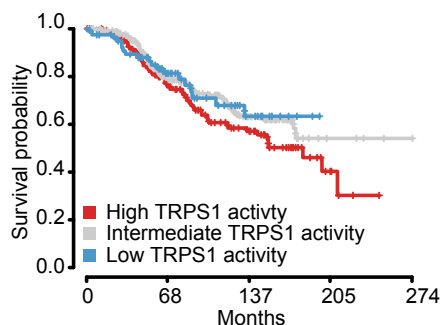**Basal-like**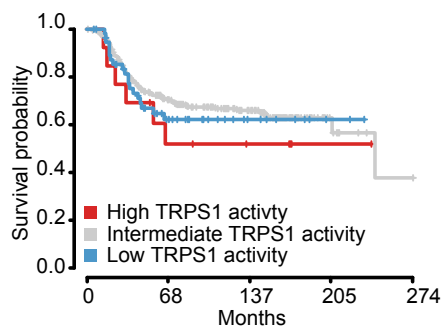**Normal-like**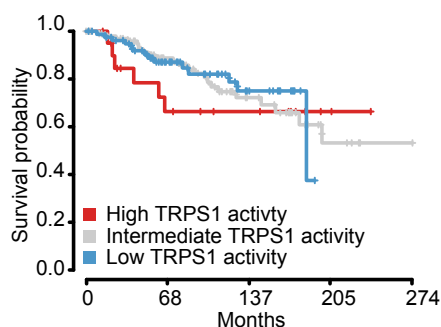**HER2-enriched**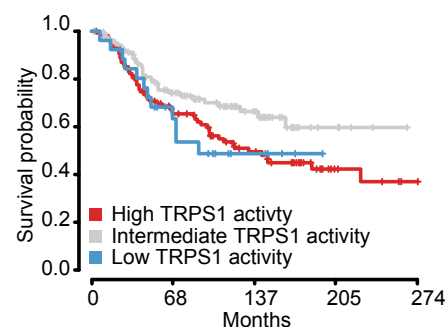

Supplement: S11 Fig — A) Kaplan-Meier curves for patients in the METABRIC cohort, stratified by TRPS1 activity as in Fig 6F, separated by ER-posivity. Logrank p-value 3.83*10-6 for ER-positive tumors and not significant for ER-negative tumors. B) Kaplan-Meier curves for patients in the METABRIC cohort, stratified by TRPS1 activity as in Fig 6F, separated by intrinsic subtype. Logrank p-value 4.23*10-4 for Luminal A tumors and not significant for the other subtypes. (PDF) [file pgen.1011159.s011.pdf]

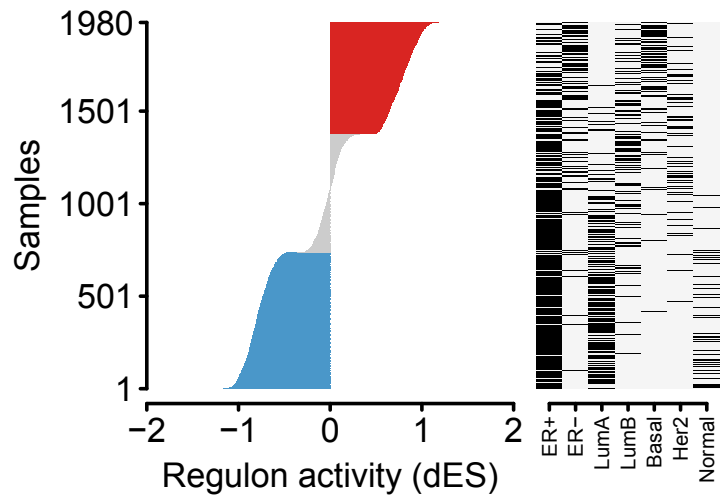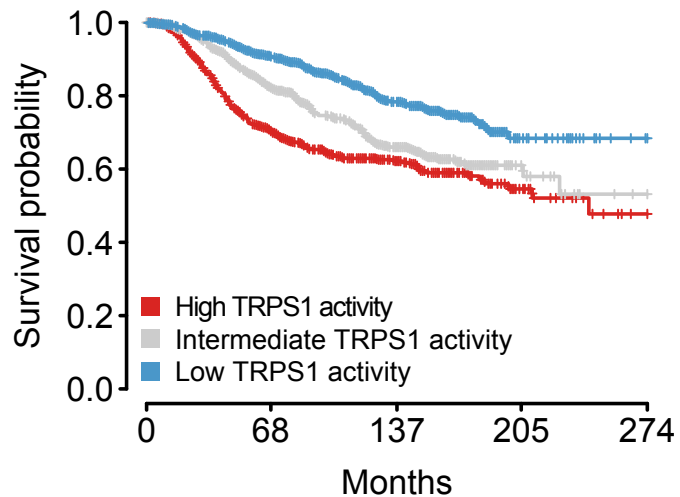

Supplement: S12 Fig — Kaplan-Meier curves for patients in the METABRIC cohort, stratified by TRPS1 activity as in Fig 6F, but using genes differentially expressed after 24 hours of TRPS1 depletion. Logrank p-value 2.09*10-13. (PDF) [file pgen.1011159.s012.pdf]
