# Supplementary material for: TRPS1 modulates chromatin accessibility to regulate estrogen receptor alpha (ER) binding and ER target gene expression in luminal breast cancer cells
Source: PLoS Genet. 2024 Feb 20;20(2):e1011159. doi: 10.1371/journal.pgen.1011159 (PMC10906895; doi:10.1371/journal.pgen.1011159)
Supplement: Attachment — Submitted filename: TRPS1_response.pdf [file pgen.1011159.s023.pdf]

Thank you to the reviewers for carefully considering this manuscript. We value their positive reception of the work and especially their constructive criticism. All the reviewers' feedback has been incorporated into the manuscript, resulting in significant improvements. We believe that the revised submission is now a more robust and compelling story, and we hope that the editor and reviewers agree.

Reviewer #1: In this manuscript, Scott et al. evaluate the role of TRPS1 in ER+ breast cancer cells. Using public data, they find that TRPS1 is important for cell growth specifically in luminal (ER+) breast cancer cells. The authors then make a T-47D line harboring an inducible degron tag on TRPS1. With this model, they find that acute loss of TRPS1 leads to reduced TRPS1 genome binding, a direct increase in chromatin accessibility at several genomic loci, and an increase in expression of genes near TRPS1 binding sites. The authors also find that TRPS1 loss rewires ER genomic binding and impacts ER target genes. Finally, the authors show that the TRPS1 target gene signature in breast cancer samples is associated with worse patient outcomes. Overall, this manuscript reports an interesting finding in a rigorous manner. The data are well described and compelling on the whole. We believe that there are a few mostly minor changes that would improve the manuscript prior to publication.

We thank the reviewer for their thoughtful suggestions for improvement of the manuscript, which we have incorporated below.

1. The PRO-seq effect in Figure 3G is very subtle and does not appear to have been tested statistically. The authors should statistically test whether PRO-seq signal is reproducibly higher with TRPS1 depletion.

Thank you for bringing this to our attention. Since composite profiles have limitations, we chose to look more closely at each TRPS1 peak and determine if increasing bidirectional transcription is a reproducible trend. We counted PRO reads in the window around the TRPS1 peak summits shown in Figure 3G and used DESeq2 to identify differentially transcribed regions. We normalized our counts based on the size factors generated from the DESeq2 analysis of the reads in genes from Figure 4. As read counts in small putative regulatory elements tend to be fewer than in large genes, it is not surprising that only 9 of these regions have individually statistically significantly higher bidirectional transcription and none with significantly lower transcription, at a false discovery rate of 0.1 (Figure S6 and below). However, we did find a TRPS1 cistrome-wide increase in PRO signal, with over 62% of these regions increasing in bidirectional transcription upon TRPS1 depletion. We performed ANOVA on a linear model predicting the logarithm of the normalized PRO reads for each region based on the DMSO or 30 minute dTAG treatment condition across the four replicates, and the p-value for the F-test was $< 2.2*10^{-16}$. The biological interpretation is consistent with our other results that indicate a role of TRPS1 in chromatin compaction. We would not necessarily expect an increase in bidirectional transcription at each TRPS1 peak upon TRPS1 degradation, but the increase in chromatin accessibility could facilitate downstream factors that promote transcription initiation. We have updated the text to reflect this analysis.

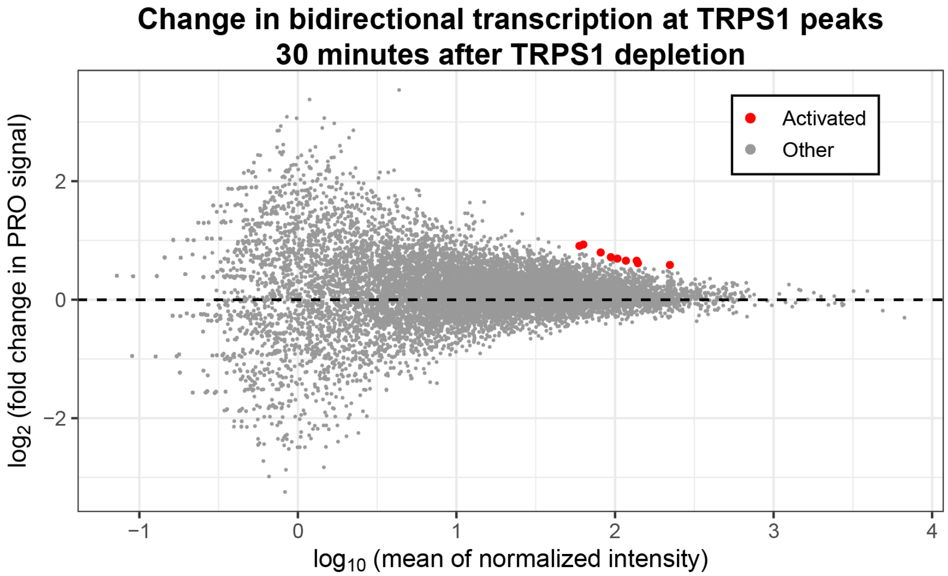

2. In the results and discussion, the authors refer to changes in ER binding or motif enrichment in ATAC sites as changes in ER activity. However, ER remains "active" and it would be more accurate to refer to this as changes in ER genomic binding or something similar.

We edited the document as follows to address this concern:

Short title (throughout): “TRPS1 modulates ER **binding and target genes**”

Abstract (page 1): “...these data suggest a model in which TRPS1 modulates the **genomic distribution** of ER…”

Introduction (page 2, line 17): “...there is a need to identify additional factors that regulate ER activity **or genomic binding**…”

Discussion (page 10, line 298): “...led us to the hypothesis that TRPS1 influences ER activity **or genomic binding**.”

Discussion (page 10, line 322): “... we identified a significant enrichment of ER half-site motifs in increased as well as decreased ATAC peaks, suggesting that ER **binding intensity** was changing in a site-specific manner.”

Discussion (page 11, line 364): “... it remains possible that TRPS1 modulates the **genomic binding intensity** of additional TFs in a unidirectional manner via a distinct mechanism. “

3. Instead of "the estrogen receptor" it should be referred to as estrogen receptor alpha, including in the title and abstract.

Thank you for pointing this out – we have implemented this suggestion.

4. For Figure 1C, the authors should clarify in the figure legend and x-axis that the black box/violin is for other cancer cell lines.

We clarified this panel (shown below).

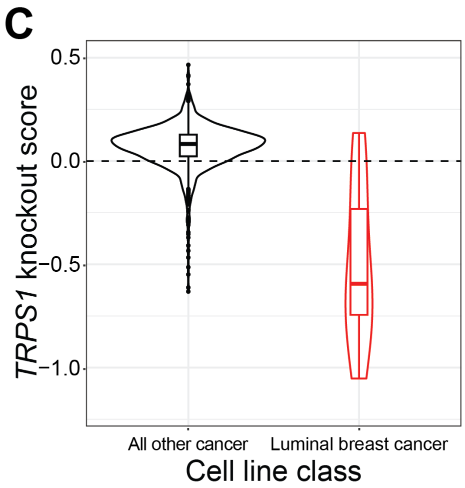

5. It would be helpful if the authors explained, possibly in the Discussion, why they chose T-47D when other breast cancer lines are more sensitive to TRPS1 KO based on Figure 1B.

We chose these cells because they are the most sensitive to *ESR1* knockout of the top four cell lines that are most sensitive to *TRPS1* knockout. We tried to tag TRPS1 in CAMA-1 and ZR-75-1 as well, but we were unable to generate clones from these cell lines. Since this is more of a practical reason and we believe that the *ESR1*/*TRPS1* dual sensitivity is sufficient justification (Figure 1B), we opted to not elaborate further in the Discussion.

6. Was the degron insertion heterozygous, the methods and results were not clear on this point? If so, why do the authors think that most of TRPS1 is removed?

The degron insertion was indeed heterozygous, and T47D cells in fact have four genomic copies of *TRPS1*. We have determined that we inadvertently created two to three knockout alleles in each of the three clones. However, we were more concerned with TRPS1 protein expression than genomic copies edited. In Figure 2A, the tagged alleles are visualized with the TRPS1 antibody, and there is no detectable untagged TRPS1. Even though we knocked out 2 or 3 alleles, we found that TRPS1 negatively regulates its own expression (TRPS1 is the most significantly activated gene upon TRPS1 depletion as measured by PRO-seq after TRPS1 degradation at any time point). This is a possible explanation for why the protein levels are comparable to the progenitor despite having fewer than 4 functional alleles. We added the following to our Methods:

"In each of the three clones, two to three of the four genomic copies of TRPS1 are knocked out, and only tagged TRPS1 protein is expressed. Details about each of the determined alleles are available at https://guertinlab.github.io/TRPS1_ER_analysis/Vignette.html#allele-sequencing."

7. The 24-hour timepoint is missing from Figure 3F.

We have modified panels 3E and F to include the 24 hour time point (shown below).

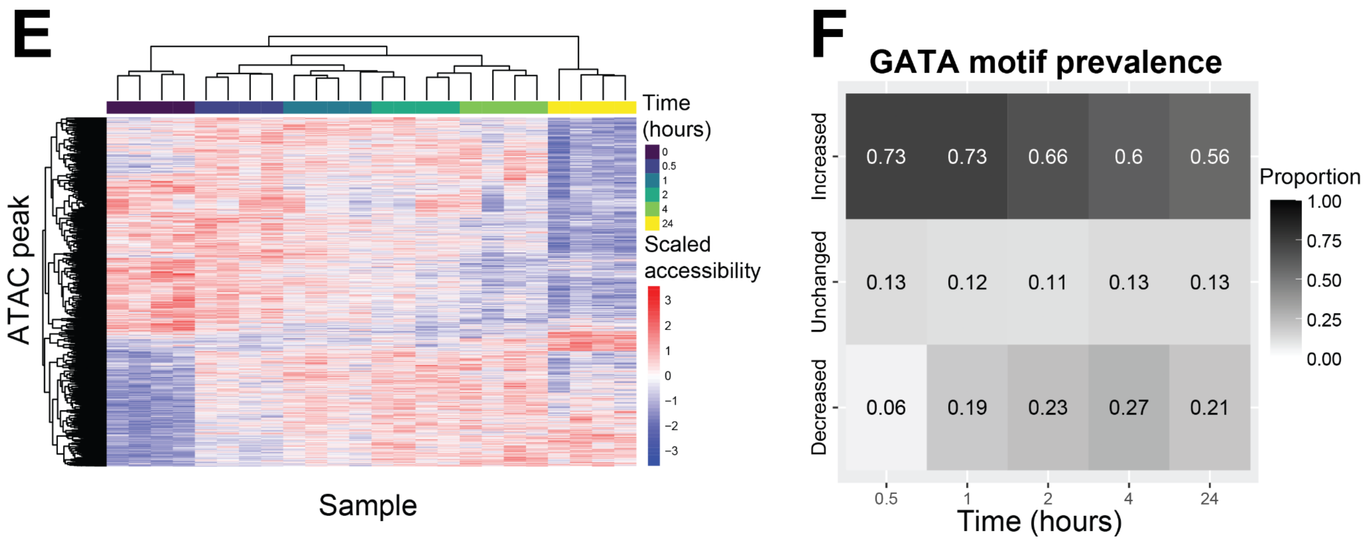

8. Ideally, Figure 6C would include a control of parental cells treated with dTAG.

Thank you for bringing our attention to the oversight of not including this important control within the manuscript. We now include a new figure showing that dTAG treatment alone does not affect growth of the isogenic parental cells (Figure S10 and below). This result strengthens our conclusion that TRPS1 depletion is responsible for the cell number defect.

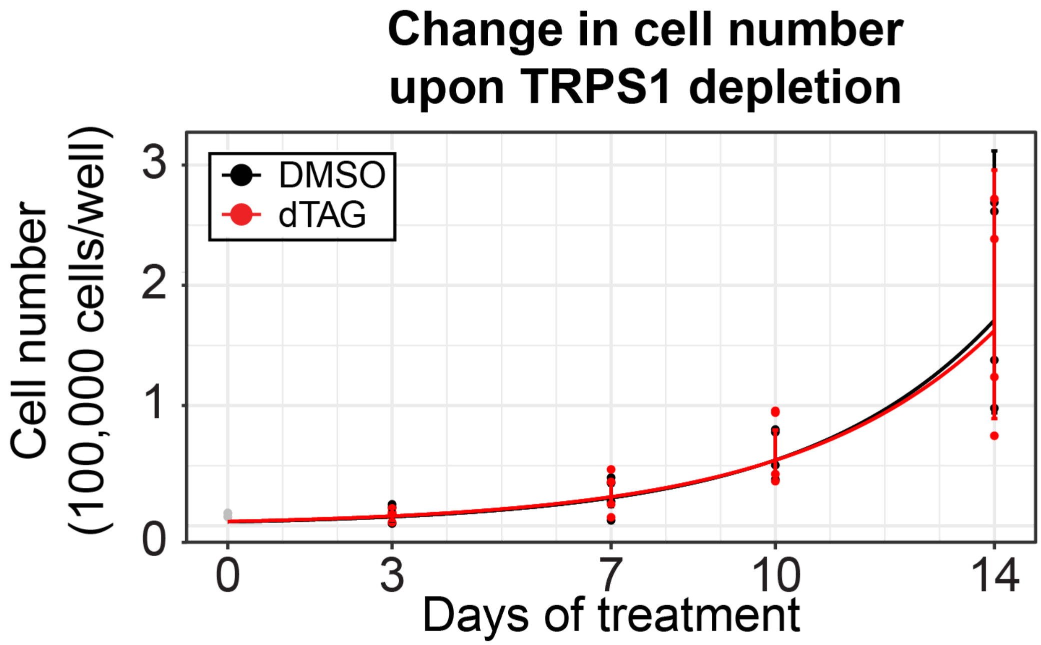

Reviewer #2: Scott et. al. used targeted protein degradation to investigate the primary effects of the GATA transcription factor TRPS1 in luminal breast cancer cells. Using a combination of TRPS1 Chip-seq, time-resolved ATAC-Seq and Pro-seq, the authors propose a model that TRPS1 directly represses the accessibility and transcription of its target genes. The authors describe that in addition to the GATA binding site, these promoters contain binding sites for the estrogen receptor (ER) and TRPS1 represses efficient binding of ER. In contrast, the authors propose an indirect activation mechanism: Genes that are downregulated after TRPS1 depletion (=TRPS1-activated) are not TRPS1 bound and are in principle accessible. Since these also contain ER binding sites, ER is bound in a TRPS1-proficient situation and transcription is active accordingly. After acute depletion, however, ER is sequestered to the previously TRPS1-bound promoters and is no longer available to the TRPS1-unbound promoters.

The manuscript is quite interesting, very clearly written and the experiments shown are valid and well presented. However, parts of the mechanism proposed here require further experimental confirmation.

We thank Dr. Wolf for his thoughtful review and positive comments. We performed additional experiments and analyses to address his concerns.

Major points:

1. a crucial aspect of this study is the direct gene regulation by TRPS1. However, no gene regulation data have been obtained in intact cells (PRO-seq measures transcription after permiabilization). In my opinion, the authors should perform Slam-seq 30 minutes and 4 hours after dTAG and analyze the regulation of old and new RNA and compare it with the Pro-Seq data. Based on this, the authors should select a set of activated and repressed genes for qPCR analysis and use this assay to examine a number of luminal (ER-sensitive and ER-in-sensitive) and ER-negative breast cancer cell lines to show that the model is universal and that the gene regulatory program is truly ER-dependent.

We agree that PRO-seq is based upon a nuclear run on assay, which requires cell permeabilization. PRO-seq directly quantifies nascent RNA from transcriptionally-competent RNA polymerases. PRO-seq also has several advantages such as detection of enhancer RNA and near single-nucleotide resolution and orientation mapping of active RNA polymerases. We believe that SLAM-seq is most useful for measuring RNA stability. While the SLAM-seq would capture newly synthesized RNA as well as PRO-seq, it is not clear what additional information this would provide.

We do not expect that the exact same genes are affected between cell lines, as indeed ER binding sites and target genes vary between cell lines, only that TRPS1 regulates ER binding at a subset of sites. As an alternative approach to address the ER-dependence of a portion of the TRPS1 effects on transcription, we have since stratified our survival analysis by ER status and by intrinsic subtype. We found that our TRPS1 activity score, based on the primary TRPS1-responsive genes, is associated with breast cancer patient survival specifically for ER-positive tumors and for Luminal A tumors, and not for ER-negative tumor types (Figure S11 and below).

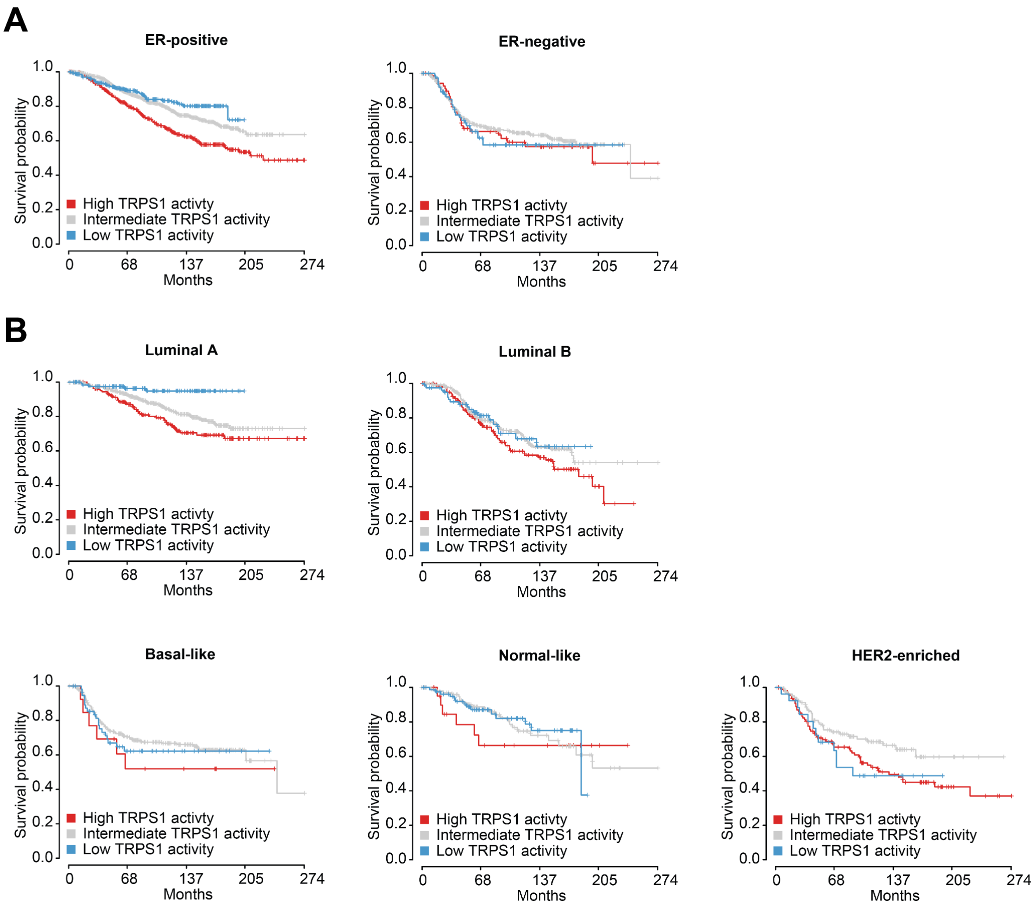

Chronic TRPS1 depletion and over-expression have many caveats, which is why we chose to focus on acute degradation and many kinetic time points upon degradation. Tagging TRPS1 in other cell lines would take many months to years, even if we only use qPCR to quantify gene expression. Since our conclusions were limited to the one T47D cell line, we have additionally performed PRO-seq in each of three independent dTAG-TRPS1 clones generated from the parental T47D cells. The genes we identified as TRPS1-regulated ER target genes in Clone 28, used for the time course experiment, tend to be regulated in the same direction upon TRPS1 degradation across three of these clones, suggesting that this effect is robust across the cell lines in which we can acutely deplete TRPS1 (Figure S9 and below).

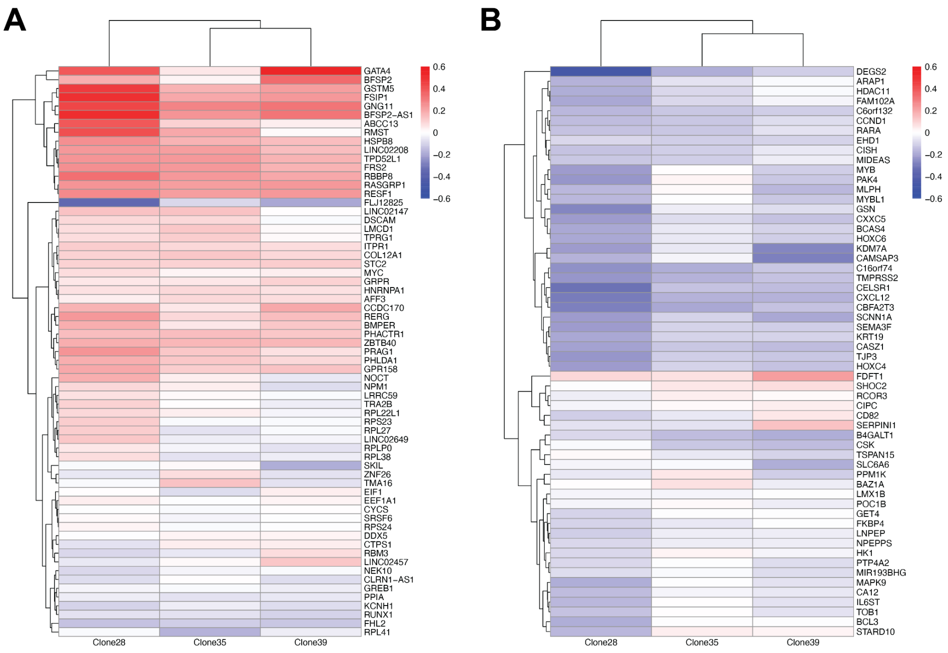

We temper our conclusions and do not suggest that this is a universal mechanism, even across all ER-positive cell lines. However, generally transcription factors tend to maintain the same molecular function between cell types. We edited the document as follows to address this concern:

Abstract (page 1): "...these data suggest a model in which TRPS1 modulates the genomic distribution of ~~other TFs~~ **ER**..."

Discussion (page 11, line 362): "While we ~~propose~~ **would predict** that our acute redistribution model is generalizable to other TFs…"

Discussion (page 12, lines 402-405): "We propose a ~~generalizable~~ model in which TRPS1 depletion leads to decondensation of local chromatin structure, allowing for the acute redistribution of ~~additional TFs like~~ ER, both activating and repressing subsets of ~~their~~ **ER** target genes."

2. the proposed indirect repression mechanism is plausible but unproven. The authors need to better substantiate this, e.g. by overexpression of ER and an associated attenuation of activation by TRPS1.

We recognize that the proposed indirect repression mechanism is difficult to prove. We agree that if overexpression of ER were to cause saturation of ER binding in the genome, then we would expect to see attenuation of activation by TRPS1. However, overexpression of a sequence-specific transcription factor does not simply saturate binding of the sites that are occupied at normal expression levels. Mass action kinetics of transcription factor binding indicates that the strongest binding sites may saturate, but the addition of more protein will result in binding at additional lower affinity sites, as has been experimentally confirmed with overexpression of MYC paralogs (PMIDs 23021215 and 29379199). Thus, even if we failed to observe attenuation of activation by TRPS1 upon overexpression of ER, then this would not invalidate our model. We also face the reality that it is impossible to overexpress ER at consistent levels across cells in a population without genetic editing and clonal expansion; transfection and transduction of nucleic acid results in a population of cells that are very heterogeneous in terms of expression.

We believe that the acute depletion experiment is the best functional validation of our model (Figure 5B,C). It is also noteworthy that we developed the hypothesis from the ATAC-seq (Figure 3C,D) and the PRO-seq data (Figure 4E,F) and tested predictions of the model by quantifying redistribution with the ChIP-seq experiment (Figure 5B,C). We would happily entertain the possibility of another model, but we cannot come up with a model that is consistent with our data. Regardless, we temper our language a bit to address Dr. Wolf's concerns. We edited the document as follows:

Author summary (page 2): "These effects on ER binding ~~lead to~~ **are associated with** changes in ER target gene ~~**activation**~~ **transcription**, ~~**attenuating or accentuating ER activation**~~ **repressing or activating these genes** in concordance with the effect on ER binding intensity."

Introduction (page 3, lines 48-49): "~~**Through**~~ **Along with** this redistribution, **we propose that** TRPS1 both directly represses and indirectly activates dozens of ER target genes at baseline."

Results (page 7, line 218): "...we focused on ER target genes to explore ~~**the**~~ **a possible** mechanism by which TRPS1 indirectly activates transcription."

Results (page 8, line 237): "~~**As we expected**~~ **Consistent with our hypothesis**, we found that ER ChIP-seq peaks…"

Discussion (page 11, lines 343-344): "...TRPS1-proximal REs increase in accessibility, ~~**allowing**~~ **an effect which we propose allows** ER to redistribute from TRPS1-distal REs. **In this proposed model,** ~~**S**~~**s**ubsets of ER target genes are activated or repressed by TRPS1 depletion via distinct mechanisms."

3. the Degron line is a great tool and should be used to phenotype acute TRPS1 loss cellularly (growth curve, cell cycle FACS, apoptosis, etc).

We generated a growth curve demonstrating a defect in cell number doubling rate upon TRPS1 loss (Figure 6C). Based on our PRO-seq data, we do not observe a transcriptional repression of cell cycle-related gene sets in the early hours after TRPS1 depletion. We do not believe that measuring cell cycle or apoptosis markers at a later time point would provide insight into a significantly earlier effect on cell cycle or apoptosis than if we were to knock down TRPS1. Indeed, TRPS1 knockdown has been shown to cause cell cycle arrest, with no change in apoptosis markers, in the ER-positive cell lines T47D (which we used in our study), MCF-7, and BT474 (PMIDs 26183398, 33691114, and 25277197). Consistent with this, we do not see evidence of apoptosis by Western blot using an antibody against cleaved-PARP1 (shown below for reviewers).

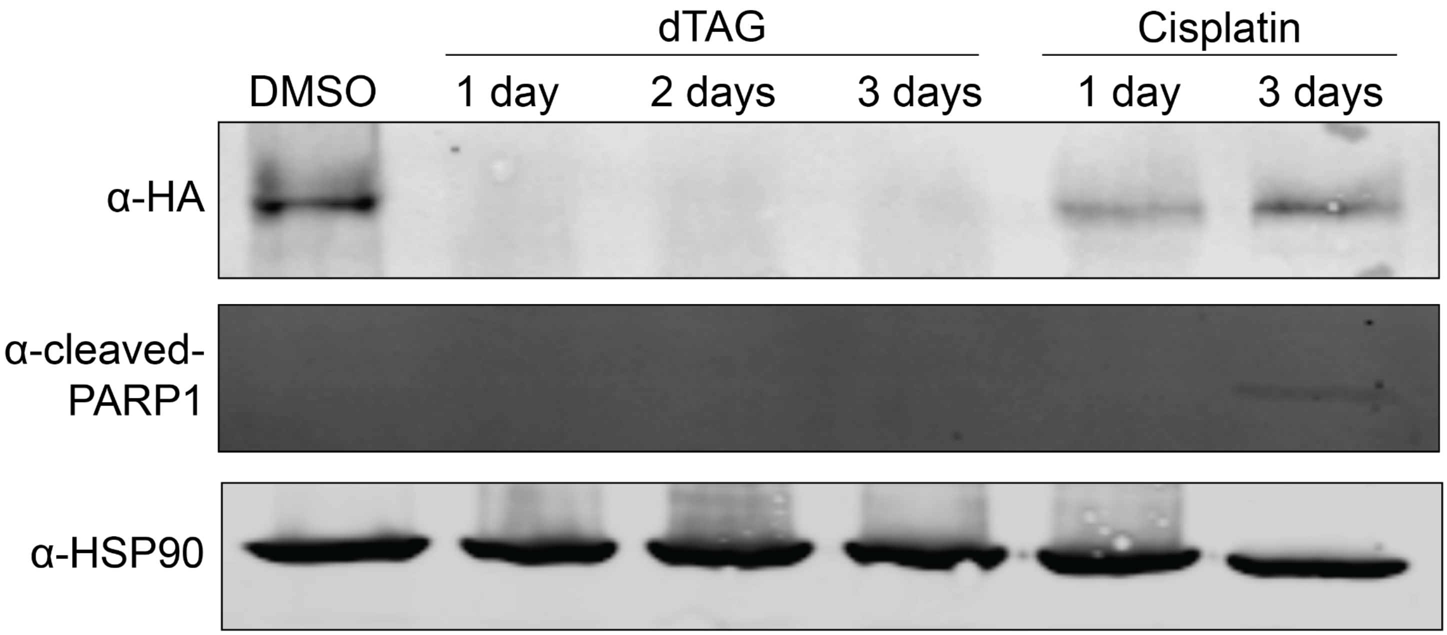

In this manuscript, we focused on assays at very short time points, for which this inducible degron cell line is the only tool available to answer our questions. There are many additional phenotyping assays that we could perform, but these cellular phenotyping experiments are not central to the claims and conclusions in this manuscript. Fortunately, we have the clones preserved and will share them with anyone who requests the cell lines.

Reviewer #3: This is a very interesting paper, clear and well written.
They chose to focus of TRPS1, a very important oncogene involved in luminal breast cancer, probably together with ER, but their functional relationships are still unclear.
They show that TRPS1 is associated with luminal breast cancer susceptibility and that luminal cancer cell lines are dependent on ER as well as on TRPS1 to proliferate.
The authors used a cutting-edge technology by integrating a degron in TRPS1 exon, so that they could induce its degradation within few minutes/hours with a chemical.
Nevertheless they also used PRO-seq to look at the real-time transcription activity.
They show that TRPS1 disappearance induces increase in chromatin accessibility together with relocation of ER at the chromatin.
The results are very convincing, all data and code are made available by the author.
This manuscript is another milestone in the road to the comprehension of ER/TRPS1 functional relations.

We thank the reviewer for their assessment of the paper and acknowledging the importance of making the data and code accessible to the community.
